# Differences in the Prevalence of Obesity, Smoking and Alcohol in the United States Nationwide Inpatient Sample and the Behavioral Risk Factor Surveillance System

**DOI:** 10.1371/journal.pone.0140165

**Published:** 2015-11-04

**Authors:** Elie S. Al Kazzi, Brandyn Lau, Tianjing Li, Eric B. Schneider, Martin A. Makary, Susan Hutfless

**Affiliations:** 1 Division of Gastroenterology and Hepatology, Department of Medicine, The Johns Hopkins University School of Medicine, Baltimore, Maryland, United States of America; 2 Department of Surgery, The Johns Hopkins University School of Medicine, Baltimore, Maryland, United States of America; 3 Division of Health Sciences Informatics, The Johns Hopkins University School of Medicine, Baltimore, Maryland, United States of America; 4 Armstrong Institute for Patient Safety and Quality, Johns Hopkins Medicine, Baltimore, Maryland, United States of America; 5 Department of Epidemiology, The Johns Hopkins Bloomberg School of Public Health, Baltimore, Maryland, United States of America; 6 Department of Health Policy and Management, The Johns Hopkins Bloomberg School of Public Health, Baltimore, Maryland, United States of America; Medical University Vienna, AUSTRIA

## Abstract

**Background:**

The lack of adequate and standardized recording of leading risk factors for morbidity and mortality in medical records have downstream effects on research based on administrative databases. The measurement of healthcare is increasingly based on risk-adjusted outcomes derived from coded comorbidities in these databases. However inaccurate or haphazard assessment of risk factors for morbidity and mortality in medical record codes can have tremendous implications for quality improvement and healthcare reform.

**Objective:**

We aimed to compare the prevalence of obesity, overweight, tobacco use and alcohol abuse of a large administrative database with a direct data collection survey.

**Materials and Methods:**

We used the International Classification of Diseases, Ninth Revision, Clinical Modification (ICD-9-CM) codes for four leading risk factors in the United States Nationwide Inpatient Sample (NIS) to compare them with a direct survey in the Behavioral Risk Factor Surveillance System (BRFSS) in 2011. After confirming normality of the risk factors, we calculated the national and state estimates and Pearson’s correlation coefficient for obesity, overweight, tobacco use and alcohol abuse between NIS and BRFSS.

**Results:**

Compared with direct participant questioning in BRFSS, NIS reported substantially lower prevalence of obesity (p<0.01), overweight (p<0.01), and alcohol abuse (p<0.01), but not tobacco use (p = 0.18). The correlation between NIS and BRFSS was 0.27 for obesity (p = 0.06), 0.09 for overweight (p = 0.55), 0.62 for tobacco use (p<0.01) and 0.40 for alcohol abuse (p<0.01).

**Conclusions:**

The prevalence of obesity, overweight, tobacco smoking and alcohol abuse based on codes is not consistent with prevalence based on direct questioning. The accuracy of these important measures of health and morbidity in databases is critical for healthcare reform policies.

## Introduction

Obesity, tobacco smoking and excessive alcohol use are leading risk factors for health complications and death in the United States (U.S.). Of the 2.5 million deaths during 2010, 9% were attributable to obesity, 18% were attributable to smoking and 4% were attributable to excessive alcohol use. In total, 750,000 deaths in 2010 were attributable to these three modifiable risk factors [1].

Despite the importance of these factors to predict health outcomes, many databases including health encounters or claims do not include information on weight, tobacco and alcohol [2]. The concept that the right data need to be included in a database to answer questions that require that data for meaningful interpretation is called “data liquidity” [3,4]. One reason that databases do not include variables or indicators of weight, tobacco smoking and alcohol use is the failure to record these factors using standard clinical coding systems like The International Classification of Diseases, Clinical Modification. However, patients are often asked to provide this information on health history forms and their height and weight is often measured by health care staff as vital signs but the information is not entered into the health encounter or claims databases resulting in incomplete recording of the information [5].

Little is known about the degree and consistency of incomplete coding of obesity, tobacco and alcohol use in administrative databases, despite tremendous enthusiasm to pay and rate hospitals based on risk-adjusted patient outcomes [4,6,7]. Commonly used risk-adjustment tools include the Elixhauser co-morbidity measure in the U.S. and the Charlson comorbidity score in both the U.S. and in the United Kingdom (U.K.). The Charlson score assigns different points for 22 medical comorbidities in order to predict one-year mortality [8]. The Elixhauser score uses 30 health comorbidities, including obesity and alcohol abuse, to predict in-hospital mortality [9]. Failure to record the variables used by the Elixhauser and Charlson measures accurately in administrative databases will result in inaccurate risk-adjustment based on these scales [10]. Outcome measures that use these risk-adjustment tools include the Patient Safety Indictors (PSI) in the U.S. [11] and the Patient Reported Outcome Measures (PROM) in the U.K. [12].

One way to examine accuracy of the information on obesity, tobacco and alcohol is to compare the prevalence of these factors using community survey data to the coded information. According to the Institute of Medicine (IOM), the best measures of obesity, overweight, smoking use and alcohol abuse in the U.S. are estimated by two Centers for Disease Control and Prevention (CDC) surveys: the National Health and Nutrition Examination Survey (NHANES) and the Behavioral Risk Factor Surveillance System (BRFSS) [13]. NHANES includes an in-person interview and health care provider measure of height and weight from around 20,000 people, and also includes statistical weights for national and regional estimates [14]. BRFSS is administered to around 500,000 people over the phone and includes self-reported height and weight that has been validated to have a high accuracy compared with health care provider measures [15]. BRFSS also includes statistical weights for state-level estimates [16].

The objective of this study was thus to compare the prevalence of obesity, overweight, tobacco smoking and alcohol abuse reported at the national and state level in the Nationwide Inpatient Sample (NIS) administrative database with direct survey using the BRFSS during 2011 to examine the accuracy of these factors.

## Materials and Methods

### Study populations and data collection

The 2011 calendar year data from BRFSS and NIS, two nationally representative de-identified databases representing direct participant survey and administrative data, were obtained. Information on body mass index (BMI), current tobacco use and current alcohol abuse from each database were compared. Both databases were de-identified and publicly available. This study was approved by the Johns Hopkins Medicine Institutional Review Board.

#### BRFSS

BRFSS is an annual survey sponsored by the CDC [16]. BRFSS collects information on the behaviors that may place the adult population (age ≥18) at risk for chronic conditions. The survey is administered during telephone interviews performed by personnel in each of the 50 states and U.S. territories. Within each state, data are collected from stratified random samples to represent the demographics of the state [16].

Height and weight used for the calculation of the BMI were self-reported by the respondent when asked “About how tall are you without shoes?” for height and “About how much do you weigh without shoes?” for weight (Table 1). Multiple questions are used to identify current smokers including: “Have you smoked at least 100 cigarettes in your entire life?” and “Do you now smoke cigarettes every day, some days, or not at all?” Current alcohol abuse included respondents with a reply of once or more in response to the question “Considering all types of alcoholic beverages, how many times during the past 30 days did you have 5 or more drinks for men or 4 or more drinks for women on an occasion?” Consistent with the nationally reported estimates [17,18], individuals with missing data for a variable were excluded from the weighted analysis for that variable [19]. Missing responses included 5.5% of BRFSS respondents for overweight and obesity, 0.5% for tobacco use and 7.3% for alcohol abuse.

**Table 1:** BRFSS questions and NIS ICD-9-CM codes used to identify the conditions of interest.

BRFSS questions	BRFSS response options	NIS ICD-9-CM codes	NIS ICD-9-CM description
**Body Mass Index (BMI)**			
“About how tall are you without shoes?” “About how much do you weigh without shoes?”	**Obesity**		
	BMI ≥ 30.00 kg/m^2^	278.00	Obesity unspecified
		278.01	Morbid obesity
		278.03	Obesity hypoventilation syndrome
		649.1	Obesity complicating pregnancy, childbirth, or the puerperium
		793.91	Image test inconclusive due to excess body fat
		V77.8^a^	Screening for obesity
		V85.30	BMI 30.0–30.9, adult
		V85.4	BMI 40.0 and above, adult
		V85.54	BMI, pediatric, greater than or equal to 95^th^ percentile for age
	**Overweight**		
	25.00 kg/m^2^ ≤ BMI > 29.00 kg/m^2^	278.02	Overweight
		V85.2	Body mass index 25.0–29.9, adult
		V85.53	Body mass index, pediatric 85^th^ percentile to less than 95^th^ percentile for age
**Tobacco use**			
Adults who are current smokers	Current smokers were considered as tobacco users, while former smokers and non-smokers were considered as non-tobacco users	305.1	Nondependent tobacco use disorder
		649.0	Tobacco use disorder complicating pregnancy, childbirth, or the puerperium
		989.84	Toxic effect of tobacco
**Alcohol abuse**			
“Considering all types of alcoholic beverages, how many times during the past 30 days did you have 5 or more drinks for men or 4 for more drinks for women on an occasion?”	Responses of ≥ 1 were considered current alcohol abuse, while non-drinkers and drinkers whose response was 0 were considered as non-alcohol abuse.	291	Alcohol-induced mental disorders
		303	Alcohol dependence syndrome
		305.0	Nondependent alcohol abuse
		790.3^a^	Excessive blood level of alcohol
		980^a^	Toxic effect of alcohol
		E860^a^	Accidental poisoning by alcohol not elsewhere classified

^a^ Additional codes that were not present in the pre-coded comorbidities in the NIS severity files.

#### NIS

NIS collects information from non-federal hospital admissions as part of the Healthcare Cost and Utilization Cost project (HCUP) sponsored by the Agency for Healthcare Research and Quality (AHRQ) [20]. NIS is the largest publically available, all-payer inpatient care database in the U.S., constituting 20% of hospital discharges from a random sample of stratified hospitals, including both academic and specialty hospitals, without regards to geographic distribution.

NIS contains discharge‐level data from approximately 8 million hospital stays during 2011 from 1,049 hospitals in 46 states [20,21].

The variables in NIS were defined using the 25 possible diagnosis positions of International Classification of Diseases, Ninth Revision, Clinical Modification (ICD-9-CM) codes. Codes for obesity, overweight, tobacco use and alcohol abuse were identified (Table 1) based on a NIS-derived comorbidity score [9,22], previous studies that used NIS [23–25] and from the list of available ICD-9-CM codes. To maintain consistency with the ages included in BRFSS, we report the results for adults aged between 18 and 99 years.

### Statistical analysis

Data management and statistical analyses were performed using the Statistical Analysis System (SAS version 9.3. SAS, Inc., Cary, NC, USA). When available, SAS code provided by BRFSS and NIS was used [26–29]. We used the Kolomogorov-Smirnov statistical test to confirm the normality of the four risk factors in both datasets. The national and state level prevalence of each condition, their correlation and the difference between the prevalence values were calculated

The national prevalence and the 95% confidence interval (CI) of obesity, overweight, tobacco use, and alcohol abuse was calculated from each data source using the appropriate sampling weights (S1 Table). The differences between the two datasets for obesity, overweight, smoking and alcohol abuse were calculated by subtracting the prevalence based on NIS from that in BRFSS. The state specific estimates were calculated for the 46 states that were represented in both databases (S2 Table).

We calculated the Pearson’s correlation coefficient of the state-level estimates for obesity, overweight, smoking and alcohol abuse between NIS and BRFSS. Statistically significant results indicate correlation (p-value < 0.05).

## Results

BRFSS included 506,467 adult participants, the weighted median age was 45.1 (range 18–99 years) and 51.3% were female. NIS included 6,828,461 adult hospitalizations, the weighted median age was 59.1 (range 18–99 years), and 50.7% were female (Table 2).

**Table 2:** Weighted BRFSS and NIS Demographic Characteristics and National Prevalence of Risk Factors, 2011.

	BRFSS	NIS	p-value
**Records in dataset**	506,467	6,828,461	-
**Median age in years (range)**	45.1 (18–99)	59.1 (18–99)	1.0
**Female, %**	51.3	50.7	1.0
**Prevalence in United States Based on Weighted Records (95% CI)**			
**Obesity, %**	27.4 (27.2–27.7)	9.6 (9.2–9.9)	<0.01
**Overweight, %**	35.8 (35.5–36.1)	0.21 (0.19–0.23)	<0.01
**Tobacco use, %**	20.1 (19.8–20.3)	12.2 (11.7–12.8)	0.18
**Alcohol abuse, %**	18.3 (18.0–18.5)	4.6 (4.3–4.8)	<0.01

Estimates based on weights provided from the BRFSS and NIS [26–29]

BRFSS, Behavioral Risk Factor Surveillance System

NIS, Nationwide Inpatient Sample

### Obesity prevalence

**Fig 1:**
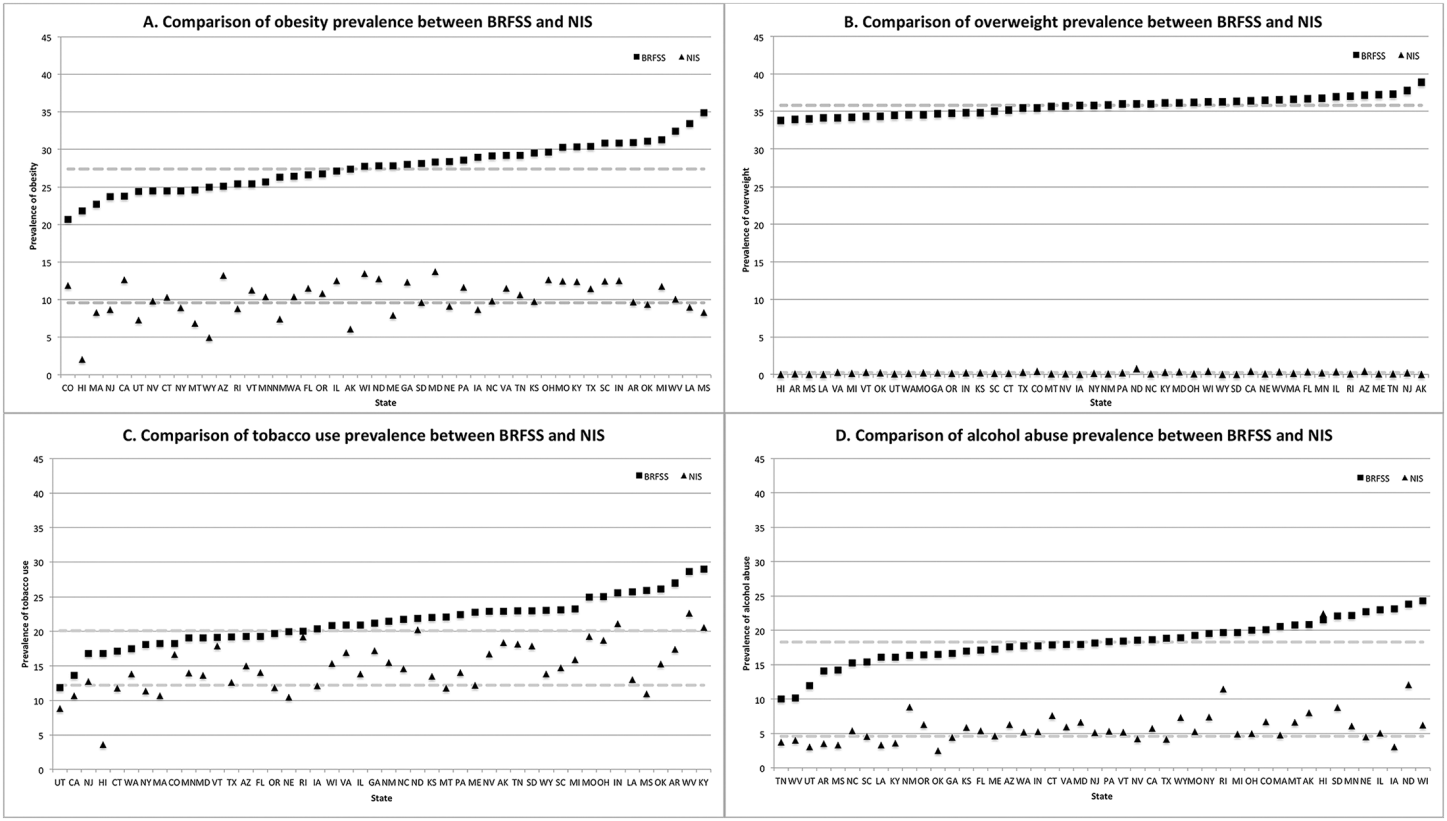
State-level comparisons between BRFSS and NIS in 2011. Dashed lines represent the national prevalence.

The prevalence of obesity was 27.4% (95% CI: 27.2%–27.7%) in BRFSS compared with 9.6% (95% CI: 9.2%–9.9%) in NIS. The correlation between BRFSS and NIS was 0.27 (p = 0.06). There was variation between the prevalence in BRFSS and NIS by state (Fig 1A). The median of the percentage point differences between the two datasets was 17.7%. Colorado had the least difference between the sources with an 8.8 percentage point difference. Mississippi had the greatest difference between the sources (26.6%).

### Overweight prevalence

The prevalence of overweight in NIS was even lower than obesity. In BRFSS, the prevalence of overweight was 35.8% (95% CI: 35.5%–36.1%) compared with 0.21% (95% CI: 0.19%–0.23%) in NIS (Fig 1B). There was no use of an overweight code among the adult population in the states of Hawaii, Wyoming and Alaska in NIS. The correlation between overweight in BRFSS and NIS was only 0.09 (p = 0.55). The median of the percentage point differences between the two datasets was 35.7%. Hawaii had the least difference between the sources with a 33.8 percentage point difference. Alaska had the greatest difference between the sources (38.9%).

### Tobacco use prevalence

The prevalence of tobacco use was 20.1% (95% CI: 19.8%–20.3%) in BRFSS compared with 12.2% (95% CI: 11.7%–12.8%) in NIS (Fig 1C). The correlation was 0.62 (p<0.01). The median of the percentage point differences between the two datasets was 6.1%. Rhode Island had the smallest difference between the sources (0.9%; BRFSS prevalence = 20.0%; NIS prevalence = 19.1%) while Mississippi had the greatest difference (15.0%).

### Alcohol abuse prevalence

The alcohol abuse prevalence was 18.3% in BRFSS (95% CI: 18.0%–18.5%), compared with 4.6% in NIS (95% CI: 4.3%–4.8%). Pearson’s correlation coefficient was 0.4 (p< 0.01) (Fig 1D). The median of percentage point differences between the two datasets was 12.6%. Hawaii had the smallest difference between the sources (-0.9%; BRFSS prevalence = 21.5%; NIS prevalence = 22.4%). Iowa had the greatest difference in prevalence between the sources (20.1%).

## Discussion

There is substantial variation in the reported prevalence of obesity, overweight, tobacco smoking and alcohol abuse between NIS, the administrative database, and BRFSS, the direct survey. After subtracting the state-level prevalence of each risk factor between BRFSS and NIS, the differences ranged between -0.9% to 35.8%. The variation is greatest for overweight where less than 1% of the U.S. population carried a diagnosis code in NIS compared with over 35% self-reporting overweight in the direct survey.

To our knowledge, this is the first study to provide a potential solution to estimate the extent to which administrative databases may be undercoding important health indicators such as weight, smoking and alcohol abuse, by comparing a U.S. administrative health dataset with a direct survey, both of which are considered to be nationally representative. The methodology and code provided to link administrative data with survey information for imputation can be used to address the gaps between these sources [5,6,23,25,30–32] until Meaningful Use or other methods of data collection are implemented [13,33–37].

The differences between NIS and BRFSS support the recommendations that researchers evaluate the accuracy of data when conducting studies and interpreting results [10,38,39]. Since NIS was made available, over 2200 publications have used the dataset as a resource (based on a PubMED query in June 2015). Many of these articles studied conditions that are associated with obesity, tobacco smoking or alcohol abuse or used the NIS recommended comorbidity score, which includes obesity and tobacco smoking as factors [9,22].

The NIS comorbidity score is also used for the risk adjustment coefficients for AHRQ’s PSI [40]. Medicare has been using PSI for hospital evaluation since 2007 [41]. A hospital’s rate of risk-adjusted outcomes has been used for payment formulas, for benchmarking the performance of different hospitals, and for public reporting of a hospital’s outcomes. Accurate coding across all hospitals is important to ensure that hospitals taking care of sicker patients are not inappropriately penalized and hospitals taking care of healthier patients are not inappropriately rewarded because of invalid risk-adjustment [42].

Improving the accuracy and the utility of information in administrative databases, like NIS will contribute to our ability to use large datasets to affect health care decisions or health policy decisions that are heavily based on the findings from these sources. A recent study found that the ICD-9-CM code for obesity was present in only 19% of those with obesity recorded in the electronic medical records [31]. Analyses that include variables that likely do not represent the true conditional state of a patient population (such as “controlling for obesity” in NIS analyses when obesity status is not recorded for a majority of obese patients), does not lead to more accurate estimates. Including mis-measured variables may even introduce further bias because the reason why individuals have a code and others do not is not known and may be meaningfully associated with the relationship under investigation.

Until accurate information on these risk factors is available in administrative databases, researchers can use direct data collection sources to adjust for the factors at the linkage level or impute missing information based on those who have complete information. This entails linking up the dataset with missing information to the accurate dataset at the most granular level of linkage possible then performing the adjustment or imputation. For our study, we linked by state since the BRFSS does not include ZIP code level estimates [16]. Another option is to collect more accurate information on height and weight, smoking and alcohol abuse in the records that contribute to administrative databases such as NIS. An approach to minimize missing data through more accurate data collection is consistent with current guidelines on the handling of missing data [43,44]. The increasing trend for electronic health records (EHRs) to include specific standardized fields for height, weight, smoking and alcohol use [45,46] could improve the comorbidity capture and consistency rate. Incorporating the fields used to record height and weight, with automatic BMI calculation, smoking status and alcohol consumption directly into the NIS system could improve the quality of information on these factors without having to use ICD codes at all [47].

EHRs compliant with Meaningful Use standards offer a unique opportunity to improve the quality of these variables large datasets. This program includes financial incentives to collect information on height, weight and smoking status as part of standard structured sets of vital signs and smoking measurements [48,49]. In addition, the “Vital Signs” report of the IOM echoes the determination of the Meaningful Use program by aligning two its 15 core measures on overweight and obesity, and addictive behavior with the efforts of adequate recording of clinical data to enhance efficiency and effectiveness of the measurements [13].

In 2013, more than 50% of all U.S. hospitals have attested to Meaningful Use programs, which would translate into more extensive collection of the smoking and height and weight measurements [50]. The alcohol abuse status is not currently required as part of Meaningful Use standards, although it is likely that substance abuse will become more integrated in the mainstream medical care and its reporting will be more prevalent in the EHRs in the near future [51] Meaningful Use will result in greater use of fields related to height, weight and smoking in EHRs [30,32,52–54].

The major strength of this study was the national representativeness of the databases compared. Each data source includes statistical weights based on the sampling technique used to ensure that the estimates will represent the U.S. population. These databases were chosen because both the national and state estimates are available. NHANES, which conducts in person interviews and measurements of participants, was not included because state level estimates cannot be calculated from the publicly available database.

Limitations of the study include the incomplete comparability of the data sources and the underestimation of obesity based of self-report in the BRFSS. NIS is strictly an inpatient database that excludes ambulatory care and emergency care whereas BRFSS surveys healthy and sick individuals sampled to represent the general U.S. population. Because obesity, overweight, smoking and alcohol abuse are associated with conditions requiring hospitalization, the true prevalence of these factors in large administrative databases like NIS may be even greater than the BRFSS prevalence. BRFSS may further underestimate the prevalence of these factors due to its reliance on self-reporting during telephone interviews [55]. For example, the 2011 national estimate of obesity is 34.9% in NHANES [56], which includes measurement of height and weight during an in-person visit, compared with 27.4% in BRFSS. If the NIS population includes individuals more likely to be overweight, obese, smokers and alcohol abuses and BRFSS underestimates these factors during self-report, then the true differences in the prevalence between sources may be even greater than those reported here.

The prevalence of obesity, overweight, tobacco smoking and alcohol abuse based on ICD-9-CM codes in an administrative database is not consistent with prevalence by direct questioning. The incorporation of Meaningful Use standard sets into NIS and other U.S. administrative databases can easily increase the accuracy of these factors without increasing the coding burden on medical personnel. Engineering a more truthful transfer of data from the health record to the database can enhance our confidence in understanding these risk factors in health care decision-making and risk-adjustment.

## Supporting Information

S1 Table SAS code and definition of data elements used to estimate the prevalence. Contains the SAS codes and the definition of the data elements that were used in the statistical analysis to estimate the prevalence of the risk factors at the national level and at the state level.
(DOCX)

S2 Table Risk factors prevalence by state. Contains the weighted state-level prevalence for obesity (Table A), overweight (Table B), tobacco use (Table C) and alcohol abuse (Table D) in BRFSS and in NIS.
(DOCX)
