# Supplementary material for: Differences in the Prevalence of Obesity, Smoking and Alcohol in the United States Nationwide Inpatient Sample and the Behavioral Risk Factor Surveillance System
Source: PLoS One. 2015 Nov 4;10(11):e0140165. doi: 10.1371/journal.pone.0140165 (PMC4633065; doi:10.1371/journal.pone.0140165)
Supplement: S1 Table — Contains the SAS codes and the definition of the data elements that were used in the statistical analysis to estimate the prevalence of the risk factors at the national level and at the state level. (DOCX) [file pone.0140165.s001.docx]

**Differences in the Prevalence of Obesity, Smoking and Alcohol in the United States Nationwide Inpatient Sample and the Behavioral Risk Factor Surveillance System**

**S1. SAS code and definition of data elements used to estimate the prevalence.**

| **SAS code for Nationwide Inpatient Sample (NIS)** (1,2) | | |
| --- | --- | --- |
| proc surveyfreq data = nis_2011;  tables agewt**VARIABLE*;  weight discwt;  strata nis_stratum;  cluster hospid;  run; | | |
| **SAS code for Behavioral Risk Factor Surveillance System (BRFSS)** (3,4) | | |
| proc surveyfreq data = brfss_2011;  tables *VARIABLE*;  weight _llcpwt;  strata _ststr;  cluster _psu;  run; | | |
| **Definition of Data elements** | | |
| **Type of data element** | **Variable** | **Coding notes** |
| **NIS 2011** | | |
| Weight | discwt | Discharge weight on Core file and Hospital Weights file for NIS. |
| Stratifier | nis_stratum | Stratum used to sample hospitals, based on geographic region, control, location/teaching status, and bed size. |
| Cluster | hospid | HCUP hospital number. |
| Domain | agewt | Age domain created to restrict the NIS dataset for adults |
| **BRFSS 2011** | | |
| Weight | _llcpwt | Final weight assigned to each respondent: Land-line and cell-phone data (Raking derived weight) |
| Stratifier | _ststr | Sample Design Stratification Variable. |
| Cluster | _psu | Primary sampling unit (Equal to Annual Sequence Number) |

**References:**

(1) Agency for Healthcare Research and Quality. 2011 NIS SAS Load Programs - Core File. 2014; Available at: [http://www.hcup-us.ahrq.gov/db/nation/nis/tools/pgms/SASLoad_NIS_2011_Core.SAS](http://www.hcup-us.ahrq.gov/db/nation/nis/tools/pgms/SASLoad_NIS_2011_Core.SAS). Accessed June/19, 2014.

(2) Agency for Healthcare Research and Quality. 2011 NIS SAS Load Programs - Hospital Weights File. 2014; Available at: [http://www.hcup-us.ahrq.gov/db/nation/nis/tools/pgms/SASLoad_NIS_2011_Hospital.SAS](http://www.hcup-us.ahrq.gov/db/nation/nis/tools/pgms/SASLoad_NIS_2011_Hospital.SAS). Accessed June/19, 2014.

(3) Centers for Disease Control and Prevention (CDC). 2011 BRFSS SAS Format Library Program. 2014; Available at: [http://www.cdc.gov/brfss/annual_data/2011/Format11.sas](http://www.cdc.gov/brfss/annual_data/2011/Format11.sas). Accessed June/19, 2014.

(4) Centers for Disease Control and Prevention (CDC). 2011 BRFSS SAS Load Program. 2014; Available at: [http://www.cdc.gov/brfss/annual_data/2011/SASOUT11_LLCP.SAS](http://www.cdc.gov/brfss/annual_data/2011/SASOUT11_LLCP.SAS). Accessed June/19, 2014.
