# Supplementary material for: Differences in the Prevalence of Obesity, Smoking and Alcohol in the United States Nationwide Inpatient Sample and the Behavioral Risk Factor Surveillance System
Source: PLoS One. 2015 Nov 4;10(11):e0140165. doi: 10.1371/journal.pone.0140165 (PMC4633065; doi:10.1371/journal.pone.0140165)
Supplement: S2 Table — Contains the weighted state-level prevalence for obesity (Table A), overweight (Table B), tobacco use (Table C) and alcohol abuse (Table D) in BRFSS and in NIS. (DOCX) [file pone.0140165.s002.docx]

**Differences in the Prevalence of Obesity, Smoking and Alcohol in the United States Nationwide Inpatient Sample and the Behavioral Risk Factor Surveillance System**

**S2 Table A. Obesity prevalence by state**

| **State** | **BRFSS prevalence** | **NIS prevalence** | **Difference**  BRFSS – NIS |
| --- | --- | --- | --- |
| Alaska (AK) | 27.4% | 6.1% | 21.3% |
| Arizona (AZ) | 25.1% | 13.3% | 11.8% |
| Arkansas (AR) | 30.9% | 9.7% | 21.2% |
| California (CA) | 23.8% | 12.7% | 11.1% |
| Colorado (CO) | 20.7% | 11.9% | 8.8% |
| Connecticut (CT) | 24.5% | 10.3% | 14.2% |
| Florida (FL) | 26.6% | 11.5% | 15.1% |
| Georgia (GA) | 28.0% | 12.4% | 15.6% |
| Hawaii (HI) | 21.8% | 2.0% | 19.8% |
| Illinois (IL) | 27.1% | 12.5% | 14.6% |
| Indiana (IN) | 30.8% | 12.5% | 18.3% |
| Iowa (IA) | 29.0% | 8.7% | 20.2% |
| Kansas (KS) | 29.6% | 9.7% | 19.8% |
| Kentucky (KY) | 30.4% | 12.4% | 18.0% |
| Louisiana (LA) | 33.4% | 9.0% | 24.5% |
| Maine (ME) | 27.8% | 8.0% | 19.9% |
| Maryland (MD) | 28.3% | 13.8% | 14.5% |
| Massachusetts (MA) | 22.7% | 8.3% | 14.4% |
| Michigan (MI) | 31.3% | 11.8% | 19.5% |
| Minnesota (MN) | 25.7% | 10.4% | 15.3% |
| Mississippi (MS) | 34.9% | 8.3% | 26.6% |
| Missouri (MO) | 30.3% | 12.5% | 17.8% |
| Montana (MT) | 24.6% | 6.8% | 17.8% |
| Nebraska (NE) | 28.4% | 9.1% | 19.3% |
| Nevada (NV) | 24.5% | 9.9% | 14.6% |
| New Jersey (NJ) | 23.7% | 8.7% | 15.0% |
| New Mexico (NM) | 26.3% | 7.4% | 18.9% |
| New York (NY) | 24.5% | 8.9% | 15.6% |
| North Carolina (NC) | 29.1% | 9.9% | 19.3% |
| North Dakota (ND) | 27.8% | 12.8% | 15.0% |
| Ohio (OH) | 29.6% | 12.7% | 17.0% |
| Oklahoma (OK) | 31.1% | 9.4% | 21.7% |
| Oregon (OR) | 26.7% | 10.8% | 15.9% |
| Pennsylvania (PA) | 28.6% | 11.7% | 16.9% |
| Rhode Island (RI) | 25.4% | 8.8% | 16.6% |
| South Carolina (SC) | 30.8% | 12.5% | 18.4% |
| South Dakota (SD) | 28.1% | 9.7% | 18.5% |
| Tennessee (TN) | 29.2% | 10.6% | 18.6% |
| Texas (TX) | 30.4% | 11.4% | 19.0% |
| Utah (UT) | 24.4% | 7.3% | 17.1% |
| Vermont (VT) | 25.4% | 11.3% | 14.1% |
| Virginia (VA) | 29.2% | 11.5% | 17.7% |
| Washington (WA) | 26.5% | 10.4% | 16.1% |
| West Virginia (WV) | 32.4% | 10.1% | 22.3% |
| Wisconsin (WI) | 27.7% | 13.5% | 14.2% |
| Wyoming (WY) | 25.0% | 4.9% | 20.1% |

**S2 Table B. Overweight prevalence by state**

| **State** | **BRFSS prevalence** | **NIS prevalence** | **Difference**  BRFSS – NIS |
| --- | --- | --- | --- |
| Alaska (AK) | 38.9% | 0.0% | 38.9% |
| Arizona (AZ) | 37.2% | 0.4% | 36.8% |
| Arkansas (AR) | 33.9% | 0.1% | 33.8% |
| California (CA) | 36.4% | 0.4% | 36.0% |
| Colorado (CO) | 35.4% | 0.4% | 35.0% |
| Connecticut (CT) | 35.2% | 0.2% | 35.0% |
| Florida (FL) | 36.7% | 0.3% | 36.3% |
| Georgia (GA) | 34.7% | 0.2% | 34.4% |
| Hawaii (HI) | 33.8% | 0.0% | 33.8% |
| Illinois (IL) | 36.9% | 0.4% | 36.5% |
| Indiana (IN) | 34.8% | 0.2% | 34.6% |
| Iowa (IA) | 35.8% | 0.0% | 35.8% |
| Kansas (KS) | 34.8% | 0.3% | 34.6% |
| Kentucky (KY) | 36.1% | 0.3% | 35.8% |
| Louisiana (LA) | 34.1% | 0.0% | 34.1% |
| Maine (ME) | 37.2% | 0.1% | 37.2% |
| Maryland (MD) | 36.1% | 0.4% | 35.7% |
| Massachusetts (MA) | 36.6% | 0.2% | 36.4% |
| Michigan (MI) | 34.2% | 0.2% | 34.0% |
| Minnesota (MN) | 36.8% | 0.2% | 36.5% |
| Mississippi (MS) | 34.0% | 0.0% | 34.0% |
| Missouri (MO) | 34.6% | 0.2% | 34.3% |
| Montana (MT) | 35.6% | 0.1% | 35.5% |
| Nebraska (NE) | 36.5% | 0.1% | 36.4% |
| Nevada (NV) | 35.7% | 0.1% | 35.6% |
| New Jersey (NJ) | 37.8% | 0.2% | 37.5% |
| New Mexico (NM) | 35.8% | 0.1% | 35.7% |
| New York (NY) | 35.8% | 0.2% | 35.7% |
| North Carolina (NC) | 36.0% | 0.1% | 35.9% |
| North Dakota (ND) | 36.0% | 0.7% | 35.2% |
| Ohio (OH) | 36.2% | 0.1% | 36.1% |
| Oklahoma (OK) | 34.3% | 0.2% | 34.1% |
| Oregon (OR) | 34.8% | 0.1% | 34.6% |
| Pennsylvania (PA) | 36.0% | 0.2% | 35.8% |
| Rhode Island (RI) | 37.1% | 0.1% | 37.0% |
| South Carolina (SC) | 35.0% | 0.1% | 34.9% |
| South Dakota (SD) | 36.3% | 0.0% | 36.3% |
| Tennessee (TN) | 37.3% | 0.1% | 37.2% |
| Texas (TX) | 35.4% | 0.3% | 35.1% |
| Utah (UT) | 34.5% | 0.1% | 34.4% |
| Vermont (VT) | 34.3% | 0.3% | 34.0% |
| Virginia (VA) | 34.1% | 0.3% | 33.9% |
| Washington (WA) | 34.5% | 0.2% | 34.4% |
| West Virginia (WV) | 36.5% | 0.3% | 36.2% |
| Wisconsin (WI) | 36.2% | 0.4% | 35.8% |
| Wyoming (WY) | 36.3% | 0.0% | 36.3% |

**S2 Table C. Tobacco use prevalence by state**

| **State** | **BRFSS prevalence** | **NIS prevalence** | **Difference**  BRFSS – NIS |
| --- | --- | --- | --- |
| Alaska (AK) | 22.9% | 18.4% | 4.5% |
| Arizona (AZ) | 19.3% | 15.0% | 4.3% |
| Arkansas (AR) | 27.0% | 17.4% | 9.6% |
| California (CA) | 13.7% | 10.7% | 3.0% |
| Colorado (CO) | 18.3% | 16.6% | 1.6% |
| Connecticut (CT) | 17.1% | 11.8% | 5.3% |
| Florida (FL) | 19.3% | 14.0% | 5.3% |
| Georgia (GA) | 21.2% | 17.2% | 4.0% |
| Hawaii (HI) | 16.8% | 3.6% | 13.2% |
| Illinois (IL) | 20.9% | 13.9% | 7.1% |
| Indiana (IN) | 25.6% | 21.1% | 4.5% |
| Iowa (IA) | 20.4% | 12.1% | 8.3% |
| Kansas (KS) | 22.0% | 13.5% | 8.5% |
| Kentucky (KY) | 29.0% | 20.6% | 8.4% |
| Louisiana (LA) | 25.7% | 13.0% | 12.7% |
| Maine (ME) | 22.8% | 12.2% | 10.6% |
| Maryland (MD) | 19.1% | 13.6% | 5.5% |
| Massachusetts (MA) | 18.2% | 10.7% | 7.6% |
| Michigan (MI) | 23.3% | 15.9% | 7.4% |
| Minnesota (MN) | 19.1% | 14.0% | 5.1% |
| Mississippi (MS) | 26.0% | 11.0% | 15.0% |
| Missouri (MO) | 25.0% | 19.3% | 5.7% |
| Montana (MT) | 22.1% | 11.8% | 10.3% |
| Nebraska (NE) | 20.0% | 10.4% | 9.5% |
| Nevada (NV) | 22.9% | 16.7% | 6.2% |
| New Jersey (NJ) | 16.8% | 12.7% | 4.1% |
| New Mexico (NM) | 21.5% | 15.5% | 6.0% |
| New York (NY) | 18.1% | 11.4% | 6.8% |
| North Carolina (NC) | 21.8% | 14.6% | 7.2% |
| North Dakota (ND) | 21.9% | 20.2% | 1.6% |
| Ohio (OH) | 25.1% | 18.7% | 6.3% |
| Oklahoma (OK) | 26.1% | 15.3% | 10.8% |
| Oregon (OR) | 19.7% | 11.9% | 7.8% |
| Pennsylvania (PA) | 22.4% | 14.0% | 8.4% |
| Rhode Island (RI) | 20.0% | 19.1% | 0.9% |
| South Carolina (SC) | 23.1% | 14.8% | 8.4% |
| South Dakota (SD) | 23.0% | 17.9% | 5.1% |
| Tennessee (TN) | 23.0% | 18.2% | 4.8% |
| Texas (TX) | 19.2% | 12.6% | 6.6% |
| Utah (UT) | 11.8% | 8.8% | 3.0% |
| Vermont (VT) | 19.1% | 17.9% | 1.2% |
| Virginia (VA) | 20.9% | 16.9% | 4.0% |
| Washington (WA) | 17.5% | 13.8% | 3.7% |
| West Virginia (WV) | 28.6% | 22.7% | 6.0% |
| Wisconsin (WI) | 20.9% | 15.4% | 5.5% |
| Wyoming (WY) | 23.0% | 13.9% | 9.2% |

**S2 Table D. Alcohol abuse prevalence by state**

| **State** | **BRFSS prevalence** | **NIS prevalence** | **Difference**  BRFSS – NIS |
| --- | --- | --- | --- |
| Alaska (AK) | 20.8% | 8.1% | 12.8% |
| Arizona (AZ) | 17.6% | 6.3% | 11.3% |
| Arkansas (AR) | 14.1% | 3.5% | 10.5% |
| California (CA) | 18.6% | 5.7% | 12.9% |
| Colorado (CO) | 20.1% | 6.7% | 13.4% |
| Connecticut (CT) | 17.9% | 7.6% | 10.2% |
| Florida (FL) | 17.1% | 5.4% | 11.7% |
| Georgia (GA) | 16.6% | 4.5% | 12.2% |
| Hawaii (HI) | 21.5% | 22.4% | -0.9% |
| Illinois (IL) | 23.0% | 5.1% | 17.9% |
| Indiana (IN) | 17.8% | 5.3% | 12.5% |
| Iowa (IA) | 23.1% | 3.1% | 20.1% |
| Kansas (KS) | 17.0% | 5.9% | 11.1% |
| Kentucky (KY) | 16.1% | 3.6% | 12.5% |
| Louisiana (LA) | 16.1% | 3.3% | 12.8% |
| Maine (ME) | 17.3% | 4.7% | 12.6% |
| Maryland (MD) | 18.0% | 6.6% | 11.4% |
| Massachusetts (MA) | 20.6% | 4.8% | 15.8% |
| Michigan (MI) | 19.7% | 4.9% | 14.8% |
| Minnesota (MN) | 22.1% | 6.1% | 16.0% |
| Mississippi (MS) | 14.2% | 3.3% | 10.9% |
| Missouri (MO) | 19.2% | 5.2% | 14.0% |
| Montana (MT) | 20.8% | 6.7% | 14.1% |
| Nebraska (NE) | 22.7% | 4.5% | 18.2% |
| Nevada (NV) | 18.6% | 4.2% | 14.3% |
| New Jersey (NJ) | 18.2% | 5.1% | 13.1% |
| New Mexico (NM) | 16.4% | 8.9% | 7.5% |
| New York (NY) | 19.6% | 7.4% | 12.1% |
| North Carolina (NC) | 15.2% | 5.4% | 9.8% |
| North Dakota (ND) | 23.8% | 12.1% | 11.7% |
| Ohio (OH) | 20.1% | 5.0% | 15.1% |
| Oklahoma (OK) | 16.5% | 2.5% | 14.0% |
| Oregon (OR) | 16.4% | 6.3% | 10.2% |
| Pennsylvania (PA) | 18.3% | 5.3% | 13.0% |
| Rhode Island (RI) | 19.6% | 11.4% | 8.2% |
| South Carolina (SC) | 15.4% | 4.6% | 10.8% |
| South Dakota (SD) | 22.1% | 8.8% | 13.3% |
| Tennessee (TN) | 10.0% | 3.7% | 6.2% |
| Texas (TX) | 18.9% | 4.2% | 14.7% |
| Utah (UT) | 12.0% | 3.0% | 8.9% |
| Vermont (VT) | 18.5% | 5.2% | 13.3% |
| Virginia (VA) | 17.9% | 6.0% | 11.9% |
| Washington (WA) | 17.8% | 5.2% | 12.6% |
| West Virginia (WV) | 10.1% | 4.0% | 6.1% |
| Wisconsin (WI) | 24.3% | 6.2% | 18.1% |
| Wyoming (WY) | 18.9% | 7.4% | 11.5% |
